# FNDC5/Irisin in dementia and cognitive impairment: update and novel perspective

**DOI:** 10.1590/1414-431X2024e13447

**Published:** 2024-07-08

**Authors:** Xiaofeng Guo, Xiaocheng Huang, Yachao Yang, Luying Dong, Dehuan Kong, Jianmei Zhang

**Affiliations:** 1 Department of Endocrinology and Metabolism, The Second School of Clinical Medicine of Binzhou Medical University, Yantai, Shandong, China; 2 Department of Health Examination, Weihai Municipal Hospital affiliated to Shandong University, Weihai, Shandong, China; 3 Department of Endocrinology and Metabolism, Weihai Municipal Hospital affiliated to Shandong University, Weihai, Shandong, China; 4 Department of Endocrinology and Metabolism, Taian City Central Hospital, Taian, Shandong, China; 5 Department of Geriatrics, Weihai Municipal Hospital Affiliated to Shandong University, Weihai, Shandong, China

**Keywords:** FDNC5/irisin, Dementia, Cognitive impairment, Alzheimer's disease, Parkinson's disease, Vascular dementia

## Abstract

Epidemiological surveys show that the incidence of age-related dementia and cognitive impairment is increasing and it has been a heavy burden for society, families, and healthcare systems, making the preservation of cognitive function in an increasingly aging population a major challenge. Exercise is beneficial for brain health, and FDNC5/irisin, a new exercise-induced myokine, is thought to be a beneficial mediator to cognitive function and plays an important role in the crosstalk between skeletal muscle and brain. This review provides a critical assessment of the recent progress in both fundamental and clinical research of FDNC5/irisin in dementia and cognitive impairment-related disorders. Furthermore, we present a novel perspective on the therapeutic effectiveness of FDNC5/irisin in alleviating these conditions.

## Introduction

Dementia is a syndrome characterized by acquired cognitive impairment, resulting in a notable deterioration in a patient’s capacity of daily living, learning, working, and socializing (1). Clinically, dementia can be classified into two categories based on its etiology: neurodegenerative conditions, which include Alzheimer's disease (AD), dementia with Lewy bodies (DLB), Parkinson's disease with dementia (PDD), and frontotemporal lobar degeneration (FTLD), and non-neurodegenerative conditions, which include vascular dementia (VaD), normal pressure hydrocephalus, as well as other conditions such as brain injury, infection, immune disorders, tumors, poisoning, and dementia resulting from metabolic diseases (1). Epidemiological surveys show that there has been a consistent increase in the prevalence of age-related cognitive impairment, AD, and dementia (2). In 2017, the global estimate for the population diagnosed with dementia was approximately 50 million and a two-fold increase is expected every two decades, resulting in an anticipated 75 million affected people by 2030 and a staggering 131.5 million by 2050 (3). Maintaining cognitive function in the aging population is a major challenge as dementia has a direct influence on the quality of life among older individuals and places considerable burden on society, families, and healthcare systems.

Exercise is believed to have a positive impact on brain health. Epidemiological research demonstrates that physical activity could reduce the risk of AD and dementia by 45 and 28%, respectively (4). Furthermore, higher levels of physical activity reduce the cognitive decline of AD, and these beneficial impacts are partially attributed to exercise's promotion of neurogenesis and synaptic plasticity, as well as the ability to reduce neuroinflammation in the adult hippocampal dentate gyrus (DG) (5). Finding out the secretory mediators that promote the positive effect of exercise on cognition holds immense potential for addressing senile cognitive decline or AD.

Recent studies have revealed that FDNC5/irisin, a novel myokine produced during exercise, has a positive impact on cognitive function and plays a crucial role in facilitating communication between skeletal muscle and the brain. In 2002, Teufel et al. (6) identified a protein called FNDC5, which contains a fibronectin type III domain and plays a role in the differentiation and development of myoblasts. Interestingly, Boström et al. (7) discovered in 2012 that an unidentified enzyme could cleave the extracellular portion of the FNDC5 protein, leading to the liberation of a polypeptide consisting of 112 amino acids into the peripheral circulation; this polypeptide was subsequently named irisin. Irisin is highly conserved in structure and is 100% homologous in mice and humans (7). Irisin is mainly secreted by skeletal muscles during exercise and it can even cross the blood-brain barrier. It has been confirmed that irisin can activate the thermogenesis of brown adipose tissue and alleviate metabolic disorders including obesity, diabetes, and cardiovascular disease (7,8). FNDC5/irisin is expressed in various tissues, such as skeletal muscle, pancreas, brown adipose tissue (BAT), liver, and brain, especially hippocampus and hypothalamus, which is closely related to its important role in memory and cognition (8).

Here, we review the updated basic and clinical research of FDNC5/irisin in dementia and cognitive impairment-related diseases and offer a novel perspective on the therapeutic value of FDNC5/irisin in these diseases.

## FNDC5/Irisin in AD

AD, the most prevalent form of dementia, is marked by a gradual decline in memory. Its pathological characteristics involve the accumulation of β amyloid protein (Aβ) plaques, nerve fiber tangles (NFTs), and oxidative stress within the brain (9). Many studies in recent years have validated the close correlation between irisin and AD (10).

### FDNC5/irisin improves cognitive function by inducing BNDF expression

BDNF (brain-derived neurotrophic factor) is widely distributed within the brain. It is critical to the survival, differentiation, migration, dendrization, and synaptogenesis of neurons, as well as hippocampal function, learning, and memory. Mutations in the *BNDF* gene leads to a decrease of BDNF secretion, leading to a volume reduction of a specific brain area, impaired situational memory function, and higher risk of anxiety and depression (11). Animal models demonstrate that exercise enhances cognitive function by boosting the expression of BDNF in different areas of the brain, such as the hippocampus (12). By blocking BDNF signaling, exercise-induced improvements in spatial learning tasks and expression of neural synaptic proteins are weakened (13).

In 2013, Wrann et al. (14) observed that the level of FNDC5/irisin in the hippocampus exhibits an increase during endurance exercise in mice. The overexpression of FNDC5 in primary cortical neurons results in an elevation of BNDF expression, whereas the knockdown of FNDC5 leads to a decrease in BNDF expression. The expression of BNDF and other neuroprotective genes in the hippocampus can be induced by augmenting circulating irisin levels through the overexpression of FDNC5 in the liver. This study demonstrated that exercise stimulated the upregulation of BDNF expression in the hippocampus via the peroxisome proliferator activated receptor gamma coactivator 1 a (PGC-1a)/FNDC5/irisin pathway, thereby exerting a beneficial influence on cognitive function.

### FNDC5/irisin improves synaptic plasticity and memory deficit

AD is distinguished by synaptic and memory impairments. A study conducted by Lourenco et al. (15) in 2019 revealed a significant decrease of FNDC5/irisin levels in the hippocampus of late-stage AD patients compared to those with early AD or individuals with normal cognitive abilities. Furthermore, AD patients exhibited lower levels of irisin in the cerebrospinal fluid (CSF) compared to patients with mild cognitive impairment (MCI) or subjects with normal cognitive function. A separate investigation revealed a positive correlation between irisin levels in the CSF of patients with AD and their scores on the Mini-Mental State Examination (MMSE), as well as the levels of Aβ42 and BNDF in the CSF (16).

In C57BL/6 mice, brain-specific knockdown of FDNC5 resulted in impaired long-term potentiation (LTP) maintenance of the hippocampus, which indicated the potential effects of FDNC5 on hippocampal synaptic plasticity and novel object recognition (NOR) memory. Administration of recombinant irisin directly into both sides of the hippocampus can protect against NOR and memory impairment caused by Aβs. C57BL/6 mice were injected intraventricularly with AdFNDC5 (an adenoviral vector overexpressing FDNC5) to elevate FNDC5/irisin mRNA and protein levels in the cortex and hippocampus. Six days later, these mice received a single lateral ventricle infusion of Aβs, and the results demonstrated that overexpression of FNDC5/irisin by injecting AdFNDC5 could effectively protect against Aβs-induced impairment of NOR and contextual fear conditioning (CFC) memory (15).

### Ablation of FNDC5/irisin affects the development/maturation of hippocampal newborn neurons and alters their morphology, transcription, and function

Islam et al. observed that providing an equivalent level of exercise to both wild-type (WT) mice and FDNC5 knockout mice (F5KO), the WT mice exhibited enhanced spatial learning and memory while the F5KO mice did not have comparable improvements in these cognitive functions. In addition, as mice aged (21-24 months), cognition of F5KO mice decreased more than that of the WT mice (17). Pattern recognition is significantly influenced by the newly generated neurons in the hippocampus (18). In aging and AD, adult hippocampal neurogenesis of mice and humans decreases (19,20), and exercise can promote neurogenesis in the adult hippocampal and improve learning and memory (21).

Abnormal activation patterns were observed in the hippocampus of adult newborn neurons in F5KO mice. Sholl analysis revealed that the dendritic complexity and overall dendritic length increased with running exercise in WT mice. As anticipated, F5KO mice did not exhibit the same growth in dendritic trees. A more complex dendrite pattern was observed in the ventral DG (dentate gyrus) of sedentary F5KO mice compared to WT mice, suggesting that the absence of FNDC5/irisin could lead to excessive growth or pruning defects of hippocampal newborn neurons (17). In addition, compared to WT mice, the spinal density of newborn neurons in the dorsal hippocampus of F5KO was significantly reduced, and the head of the dendritic spine was smaller (17). According to some studies, the dorsal hippocampus controls cognitive functions, whereas the ventral hippocampus controls emotional behaviors (22). These findings indicate that FNDC5/irisin has a specific impact on the growth and maturation of new neurons in the hippocampus, consequently influencing cognitive abilities.

FNDC5/irisin not only affects the development/maturation of hippocampal newborn neurons, but also changes their transcriptome. Regardless of exercise intervention, the transcriptional profile of newborn neurons in F5KO mice was significantly abnormal. RNA sequencing of the nucleus showed more variability, revealing a total of 459 genes expressed differently between F5KO and WT newborn neurons. Gene enrichment analysis showed that the knockdown of FNDC5/irisin in hippocampal newborn neurons could lead to a series of major diseases, including AD, similar to “neurodevelopmental interruption or neurotrophic signal transduction disorders” (17).

The RNA sequencing data obtained from the Mount Sinai School of Medicine and Mayo (MSSM) study, comprising a total of 2114 samples derived from 1234 individuals, showed a significant decrease in FNDC5 expression in the parahippocampal gyrus of individuals diagnosed with AD compared to the control group (17). In APP/PS1 mice, a well-known transgenic AD mice model, FNDC5 gene expression in the hippocampus showed a notable decrease compared to WT mice, and when they reached 6 months of age, APP/PS1 mice initiated the formation of amyloid plaques, experienced gliosis, and exhibited cognitive decline. The cognitive function of APP/PS1-F5KO mice (generated by hybridization of F5KO mice and APP/PS1 mice) was decreased following exercise compared to APP/PS1-WT mice. Furthermore, APP/PS1-F5KO mice displayed significantly increased levels of soluble Aβ-40 in the cortex, thereby promoting the formation of Aβ plaques. Male APP/PS1 mice aged 8 months with overexpression of irisin in the liver and increased circulating irisin to pharmacological levels, despite the absence of irisin overexpression in the hippocampus, exhibited significant improvements in spatial learning and memory tasks when they reached the age of 10 months. 5xFAD mice, another type of transgenic AD mice, upon receiving treatment with AAV8 irisin, showed an improvement in spatial learning and memory performance during the Morris water maze (MWM) test (23). These results suggest that the peripheral administration of irisin enhances cognitive abilities in animal models of AD.

In conclusion, FNDC5/irisin induces BNDF expression and improves the synaptic plasticity and memory deficit of hippocampal neurons in the AD model. In addition, it increases the development/maturation of hippocampal newborn neurons and changes their morphology, transcription, and function, thus playing a pivotal role in the onset and advancement of AD. Moreover, the above studies show that the administration of irisin through peripheral means effectively mitigated the deterioration of cognitive function, even if substantial pathological changes of AD had occurred in the mouse brain (23). Therefore, there is compelling evidence to advocate for the utilization of irisin as a novel therapeutic intervention for Alzheimer's disease.

## FNDC5/Irisin in Parkinson's disease with dementia (PDD) and dementia with Lewy bodies (DLB)

PD is considered the second most prevalent neurodegenerative disorder after AD (24). The pathological characteristics of PD include the loss of dopaminergic neurons in the substantia nigra pars compacta (SNpc), the aggregation of a-synuclein (a-syn), mitochondrial and lysosomal dysfunction, abnormal synaptic transmission, and neuroinflammation (25). In addition to motor symptoms like stiffness, tremor, and gait disorders, PD also leads to non-motor symptoms including constipation, orthostatic hypotension, rapid eye movement sleep disorder, depression, and dementia (26). It is common for PD patients to suffer from dementia, and the time of dementia onset determines how patients are classified. Patients who develop dementia before parkinsonism, or during the first year of PD are classified as DLB. Those patients in whom dementia develops more than one year after the onset of motor signs are defined as PD with dementia (PDD). These two diseases share the same core clinical features and pathogenesis. Both PDD and DLB are categorized as synucleinopathies due to the existence of Lewy bodies and Lewy neurites, resulting from the accumulation of α-synuclein (α-syn), which give their distinctive pathological characteristics (27).

Some studies have found that physical exercise can reduce posture and gait instability in PD patients, improve overall mobility, and boost cognitive abilities including processing speed and cognitive control (28,29). However, the precise molecular mechanisms responsible for the benefits of physical activity in PD have yet to be fully elucidated. There are some studies indicating that the myokine irisin induced during exercise may be the beneficial mediator and may serve as a future treatment for PD (30).

### Irisin prevents the formation of pathologic **α**-syn and protects neurons against **α**-syn-induced neurotoxicity

The propagation of pathologic α-syn in the brain of patients with PD is believed to occur in a manner similar to prion transmission. This process ultimately results in neuronal dysfunction and death. When primary cortical neurons were cultured *in vitro* and exposed to α-syn PFF (α-synuclein preformed fibril), endogenous misfolding of α-syn occurred, which was subsequently toxic to cells (31). During treatment of cortical neurons with α-syn PFF, treatment with irisin at a concentration of 5 ng/mL significantly reduced pathological α-syn, and 50 and 500 ng/mL irisin prevented the formation of syn and cortical neuron death induced by α-syn PFF (32).

The results of an injection of α-syn PFF into the striatum of mice and a subsequent injection two weeks later of AAV8-irisin via the tail vein to induce overexpression of irisin in the liver and circulation (control group was AAV8 GFP) showed that the injection of AAV8 GFP resulted in a 60% loss of DA neurons, while injection of AAV8 irisin resulted in only a 25% loss. Tyrosine hydroxylase (TH) levels and expression of dopamine transporter (DAT) decreased by 49 and 45%, respectively, while with injection of AAV8 irisin only, TH levels decreased by 6%. Importantly, compared to mice treated with AAV8 GFP, the administration of AAV8 irisin effectively inhibited the aggregation of insoluble α-syn and significantly ameliorated the behavioral impairments induced by α-syn PFF (32).

### Irisin exhibits a protective effect against apoptosis of dopaminergic neurons

The progressive deterioration of dopamine neurons in the substantia nigra pars compacta (SNpc) is a hallmark characteristic of PD. A PD rat model was established by Zarbakhsh et al. (33) through intranasal administration of the toxin MPTP (1-methyl-4-phenyl-1,2,3,6-tetrahydropyridine). The PD rats were treated with bone marrow stem cells (BMSCs), irisin, or BMSCs combined with irisin, and the findings indicated that compared to the control group, MPTP significantly reduced the apoptosis of dopaminergic neurons by 77%. However, in the groups treated with BMSCs, irisin, and irisin+BMSCs, neuronal damage was 63, 56, and 46%, respectively. In addition, compared with the PD group, all treatment groups showed significant preservation of TH+ cells (tyrosine hydroxylase-positive neurons). However, there was a lack of a statistically significant difference between the irisin group and the BMSCs group, so it was postulated that irisin could promote stem cell migration to SNpc and transform them into dopaminergic neurons. Subsequently, apoptosis was assessed, and the proportion of apoptotic cells in the SNpc region of the control and MPTP groups was 20.62± 0.9 and 62.76±1.6%, respectively, 46.8±8% in the BMSCs group, 39.4±1.1% in the irisin group, and 28.6± 1.5% in the irisin+ BMSCs group. The irisin combined with BMSCs group exhibited the highest degree of reduction, while no statistically significant difference was observed between the irisin group and the BMSCs group (34).

### Irisin prevents mitochondrial damage in PD

Increasing evidence suggests that mitochondrial dysfunction plays a crucial role in PD etiology (35). A recent study showed that after 12 weeks of regular exercise, serum concentration of irisin in PD patients increased notably, and their exercise capacity and balance functionality improved. Additionally, the administration of irisin enhanced motor function and mitigated dopaminergic neurodegeneration in MPTP-induced PD mice, while concurrently reducing apoptosis of dopaminergic neurons (36). Subsequent investigations revealed that irisin, through the activation of the ERK1/2 (extracellular signal regulated kinase 1/2) signaling pathway, effectively mitigated intracellular oxidative stress, suppressed mitochondrial rupture, and facilitated mitochondrial respiration and biogenesis in PD models, ultimately leading to a reduction in neuronal apoptosis (36).

In summary, irisin demonstrates neuroprotective properties in PD by inhibiting the formation and accumulation of pathogenic α-syn, mitigating the apoptosis of dopaminergic neurons, and alleviating mitochondrial impairment.

## FNDC5/Irisin in vascular dementia (VaD)

VaD is a cognitive disorder resulting from the diminished cerebral blood flow caused by dysfunction of the cerebral vascular system. Chronic cerebral hypoperfusion (CCH) is a fundamental pathophysiological feature of VaD, as long term ischemia causes white matter lesions (WML) and hippocampal atrophy, resulting in cognitive impairment and memory loss through complex molecular and pathway mechanisms. In severe cases, patients may also experience mental disorders, such as depression, anxiety, and attention and executive dysfunction (37). In recent years, there has been a notable increase in the incidence of VaD (38). Tu et al. (39) reported that a decrease of irisin concentration was associated with poor functional outcomes in patients with ischemic stroke. Zhang et al. (40) observed a significant reduction in serum irisin levels in patients with VaD compared to the control group. After adjusting for all clinical features, results of Spearman analysis and logistic regression showed a significant positive correlation between irisin levels and Montreal Cognitive Assessment (MoCA) scores in patients with VaD.

### Irisin reduces hippocampal neuron apoptosis and local inflammation in an ischemia/reperfusion mouse model through multiple signaling pathways

An ischemia/reperfusion (I/R) mouse model was created through the surgical ligation of the bilateral common carotid arteries (BCCA) for a duration of 20 min, followed by a 24-h reperfusion. The neurological deficiency scale (NDS) showed that the I/R mice had severe neurological deficits, while administration of irisin led to a significant reduction in the NDS score. *In vivo* and *in vitro*, irisin reduced apoptosis of hippocampal neurons. In addition, irisin significantly inhibited the expression of inflammatory cytokines, such as interleukin 1β (IL-1β) and tumor necrosis factor α (TNF-α), while simultaneously upregulating the expression of Notch1 intracellular domain (NICD), Notch1, and Hes1. These findings suggest that irisin exerts a neuroprotective effect on I/R injury by modulating the Notch signaling pathway (41).

In the chronic cerebral hypoperfusion (CCH) VaD mouse model established by bilateral common carotid artery stenosis (BCAS), FNDC5/irisin levels in the hippocampus of BCAS mice significantly decreased, and overexpression of FNDC5 in the hippocampus or injection of recombinant irisin into the bilateral hippocampus improved the synaptic plasticity and alleviated hippocampus inflammation, thereby reducing cognitive impairment of BCAS mice (42).

In another study using a mouse stroke model, which was established by occlusion of the middle cerebral artery (MCAO) for 45 min and reperfusion for 23 h, intracerebroventricular (ICV) injection of irisin at doses of 0.1, 0.5, 2.5, 7.5, and 15 µg/kg was performed at the beginning of MCAO. The results showed that irisin significantly reduced the infarct area, but only the 7.5 and 15 µg/kg doses improved clinical outcomes. Irisin (7.5 µg/kg) treatment alleviated brain edema, significantly reduced apoptotic cells in ischemic cerebral cortex, and increased the immunoreactivity of BDNF, but the permeability of the blood brain barrier was unchanged (43).

Moreover, Li et al. (44) found a negative association between plasma irisin level and cerebral infarction volume, neurological deficit score, and TNF-α and IL-6 (interleukin 6) plasma concentrations. Administration of irisin resulted in an elevation in the phosphorylation levels of Akt and ERK1/2, and conversely, inhibition of Akt and ERK1/2 attenuated the neuroprotective properties of irisin. Consequently, the activation of the Akt (serine and threonine-specific protein kinase) and ERK1/2 signaling pathways by irisin may be a mechanism through which neurons are safeguarded against I/R-induced damage.

### Irisin improved cognition in cerebral ischemia mouse model by regulating Klotho expression

The *Klotho* gene serves as a regulator of aging (45), with its associated protein playing a crucial role in the delay of aging and the enhancement of cognition (46). In mice with *Klotho* mutations, lifespan was shortened, synaptic integrity was impaired, and cognitive function was compromised (47). Some clinical studies have found a significant decrease of *Klotho* concentration in the CSF among elderly individuals compared to their younger counterparts. Moreover, these studies also found a strong correlation between *Klotho* mutations and the onset of cognitive dysfunction in the elderly population (48). In addition, a notable decrease in the concentration of Klotho protein was observed in the CSF of individuals diagnosed with AD (49).

Jin et al. (50) found a significant positive association between irisin levels and Klotho concentrations in CSF of stroke patients, and CSF irisin levels and MoCA scores were positively correlated. In the MCAO stroke mouse model, both physical exercise and exogenous irisin demonstrated comparable neuroprotective effects on cognitive impairment. In comparison to the MCAO group, the irisin treatment group exhibited a notable increase in the expression of Klotho protein, as well as forkhead transcription factor (FOXO3a) and manganese superoxide dismutase (MnSOD). Additionally, DHE staining demonstrated a reduction in the formation of reactive oxygen species (ROS). Subsequent investigations revealed that these protective effects of irisin disappeared in *Klotho* knockout mice. These findings indicate that irisin mitigated oxidative stress by regulating Klotho expression, consequently improving cognitive function and clinical outcomes in the cerebral ischemia mouse model.

In summary, serum and CSF irisin are positively correlated with cognitive function in patients with vascular dementia. In different animal models of VaD, irisin alleviates inflammation and oxidative stress levels by regulating Notch and Akt/ERK1/2 signaling pathways, and Klotho protein protects neurons from apoptosis, thereby attenuating cognitive dysfunction in vascular dementia.

## FNDC5/Irisin in cognitive impairment and dementia caused by depression

Evidence from clinical and epidemiological studies has demonstrated that elderly individuals with a prior history of depression are at a higher risk for developing MCI and dementia (51,52). In a recent longitudinal study encompassing a population of 1.7 million New Zealand citizens over a span of 30 years, a significant association was discovered between prior occurrences of mental disorders and the onset of dementia. This extensive population-based study provided further support for the notion that emotional disorders were strongly associated with dementia (53). Moreover, the elderly population exhibited a high prevalence of depression, which frequently manifested as a complication of dementia (51,54). Depression is characterized by various physiological processes, such as vascular disease, alterations in glucocorticoid signal transduction, hippocampal atrophy, brain inflammation, and deficiencies in BDNF (51). There has been a notable rise in the prevalence of depression in recent years, which has emerged as a significant global health concern. Studies have demonstrated that physical exercise is beneficial for mental well-being, particularly anxiety and depression, and it is suitable for all age groups encompassing children, adults, and the elderly (55,56). As irisin is a myokine induced by motor activity and has been identified as playing a significant role in neurological and cognitive disorders, many studies had been undertaken to examine the correlation between irisin and depression, as well as depression-related dementia.

### Reduction in central FNDC5/irisin levels may represent a shared pathological mechanism between major depressive disorder (MDD) and AD

A previous study involved 63 elderly subjects (38 individuals had cognitive impairment and 25 individuals did not have cognitive impairment) from which blood and CSF were collected. The 15-item Elderly Depression Scale (GDS-15) was used to screen 39 depression symptoms. The findings indicated that 41 elderly individuals were diagnosed with depression, which was associated with a decrease of irisin and BDNF levels in CSF, comparable to the observed outcomes in dementia patients (57). MDD served as both a comorbidity and a predisposing factor for the onset of AD (58). Regular physical exercise was associated with reduced incidence and severity of MDD and AD (59). Lima-Filho et al. (60) assessed the expression of FNDC5 in postmortem brain tissue obtained from a mature cohort of individuals diagnosed with MDD and MDD with psychotic features (MDD-P), as well as healthy control subjects, sourced from the Stanley Medical Research Institute brain bank. They found that the expression of FNDC5 mRNA in the dorsolateral prefrontal cortex of both MDD and MDD-P patients exhibited a notable decrease compared to the control subjects, and no discernible gender-based differences were observed. Similar results were also obtained in their MDD mouse model. Moreover, FNDC5 expression declined in the frontal cortex of male mice with lipopolysaccharide-induced depression, while it was unaltered in the hippocampus. The study also revealed that social isolation did not elicit any alterations in the expression of FNDC5 in the frontal cortex or hippocampus of mice. According to these findings, FNDC5 exerted a regionally specific regulatory effect on depressive behavior, and the decline of central FNDC5/irisin may represent a shared pathological mechanism between MDD and AD (60).

### Antidepressant effects of irisin

The study conducted by Pignataro et al. (61) revealed that administration of irisin by subcutaneous injection at a dose of 100 µg/kg per day for 5 days resulted in notable antidepressant and anti-anxiety effects in juvenile mice, without any observable gender-based distinctions.

Postoperative depression is a topic of significant concern in the academic community. Previous studies have indicated that administration of propofol resulted in depressive symptoms in mice, and irisin has been shown to have a significant positive impact on the depressive behavior induced by propofol. Furthermore, irisin mitigated neuronal death caused by high propofol concentrations *in vitro* and inhibited propofol-induced cytokine elevations in astrocyte cultures (62). EGFR (epidermal growth factor receptor) has been found to be correlated with the occurrence of depression in individuals diagnosed with severe MDD (63), and a significant increase in EGFR expression was observed in mice treated with propofol, which can be effectively inhibited by irisin (62). In the rat depression model induced by chronic unpredictable stress (CUS), administration of irisin resulted in a significant increase in glucose transport and phosphorylation levels. These findings suggest that irisin may have antidepressant-like effects in CUS rats by modulating energy metabolism in the prefrontal cortex of the brain (64).

In brief, research findings suggest that a decrease in central FNDC5/irisin may be a common pathological mechanism underlying both depression and AD. Animal studies have demonstrated that administration of irisin can alleviate depressive and anxious behaviors, indicating its potential as a future therapeutic intervention for depression and dementia.

## FNDC5/Irisin in cognitive impairment and dementia caused by diabetes

Diabetes is the most common metabolic disorder, and it has been proven that diabetes increases the risk of cognitive decline, especially in working memory, information processing speed, and executive function (65). Epidemiological studies have shown a correlation risk ratio ranging from 1.43 to 1.62 between diabetes and dementia (66,67). A hierarchical relationship was observed between the age of onset of type 2 diabetes and dementia: the younger the onset age of diabetes, the higher the risk of dementia later (68). The identification of structural anomalies such as cerebral atrophy in the brain of individuals with diabetes provides evidence for the epidemiological association between diabetes and dementia, which is usually related to cognitive decline (69). Moreover, a strong correlation was found between diabetes and other predisposing factors of dementia, including hypertension and atherosclerosis (65). The role of irisin in diabetes-related cognitive impairment has also received extensive attention.

A clinical investigation was conducted in a cohort of 133 individuals who had been diagnosed with diabetes, consisting of 59 patients with MCI and 74 patients without cognitive impairment serving as control subjects. The findings of the study revealed that MCI diabetes patients demonstrated increased levels of irisin in their plasma and more pronounced insulin resistance compared to their cognitively healthy counterparts. Elevated concentrations of plasma irisin have been associated with impaired cognitive abilities, particularly in the executive function domain. Additionally, linear regression analysis demonstrated a correlation between irisin and glycated hemoglobin, and both were independent risk factors for MCI in diabetes (70).

Administration of irisin via subcutaneous injection enhances the cognitive function of diabetic mice induced by streptozotocin (STZ). This effect may be attributed to the inhibition of astrocyte activation, the reduction of hippocampal presynaptic vesicle protein synaptophysin (SYP), and the alleviation of neuroinflammation through the reduction of IL-1β and IL-6 levels in the brain of DM mice (71). Furthermore, in SD rats with diabetes induced by a combination of STZ and high-fat diet, a reduction of serum BDNF and irisin was observed, and subcutaneous administration of irisin enhanced the expression of BDNF in the hippocampus of diabetic rats and adversely affected the levels of serum GHbA1c and AGEs. The findings of this study suggest that irisin may have a significant impact on cognitive function regulation in rats with type 2 diabetes, achieved through the modulation of BDNF expression and glucose metabolism (72).

However, the role of irisin levels in the CSF of individuals with diabetes-related cognitive impairment remains unclear and should be further studied. Furthermore, the potential involvement of irisin in other endocrine and metabolic disorders, such as cognitive impairment and dementia associated with hypothyroidism, has yet to be investigated.

## FNDC5/Irisin in cognitive impairment and dementia caused by brain injury

Acute brain injury (ABI) includes stroke and traumatic brain injury (TBI). Cognitive impairment is a prevalent and disabling outcome of TBI. Mild traumatic brain injury (mTBI), commonly referred to as concussion, is characterized by a minor head injury resulting in a transient loss of consciousness, subsequently leading to cognitive impairment, which represents the most frequently encountered form of TBI (73,74). mTBI has been found to have a detrimental impact on various cognitive domains, such as executive function, learning and memory, attention, and processing speed (75). In a 1-year prospective cohort study comprising 656 individuals diagnosed with mTBI and aged over 17 years, it was observed that 13.5% of the mTBI participants exhibited suboptimal cognitive outcomes at the end of the follow-up period; this percentage was notably higher compared to the control group, in which only 4.5% demonstrated similar cognitive impairments (73). Delaplain et al. (74) found that a significant proportion of adult trauma patients diagnosed with intracranial hemorrhage (ICH) and mTBI displayed cognitive impairment during the initial phases. Similar results were obtained by Keys et al. (76) in their study of a pediatric TBI cohort. It has been shown that brain injury could result in a series of interconnected events and pathological processes, including energy consumption disorder, cell death, mitochondrial dysfunction, inflammation, free radical generation, oxidative stress, and apoptosis (77). Extensive studies and investigations have provided substantial validation for the ability of irisin to enhance neurogenesis, cell proliferation, and neural synaptic plasticity in embryonic stem cells (ESCs) (78,79). Importantly, irisin has been demonstrated to exert a crucial role in mitigating inflammation, alleviating oxidative stress, mitigating cell apoptosis, and enhancing impaired mitochondrial function (80). Consequently, the significance of irisin in the context of TBI is progressively being recognized.

### Irisin mitigates mitochondrial dysfunction via the PGC1**α**-dependent pathway

During the acute phase of brain injury, there is a pressing requirement for an adequate supply of adenosine triphosphate (ATP) to facilitate the repair of damaged cells (81). However, the impaired mitochondria lose the capacity to fulfill this energy requirement, which triggers a series of detrimental cascading reactions, including malfunctioning of the electron transport chain, depletion of ATP, excessive generation of reactive oxygen species (ROS), injury caused by oxidative stress, neuronal apoptosis, and neurogenic inflammation (82). Recent *in vivo* and *in vitro* studies have underscored the significant role of irisin in preserving mitochondrial function and promoting mitochondrial biogenesis. Treatment with exogenous irisin has been observed to mitigate mitochondrial dysfunction, leading to enhanced ATP utilization (83,84). Fan et al. (83) revealed that irisin impeded the formation of free radicals and the escalation of inflammatory factors. Furthermore, the administration of irisin has been found to partially sustain mitochondrial potential and cellular ATP levels through the involvement of AMPK-dependent pathways (79). Irisin treatment could stimulate mitochondrial biogenesis and inhibit mitochondrial division, thereby compensating for excessive ATP consumption (84).

### Irisin alleviates acute brain injury by increasing BNDF levels

In clinical practice, levels of BDNF have been used as biomarkers for predicting mortality and outcomes after brain injury (85). BNDF plays several neuroprotective roles, including promoting nerve regeneration and adult neurogenesis, mediating synaptogenesis and synaptic plasticity, as well as enhancing cell survival and mitigating apoptosis of neurocytes (86). Therefore, it can be inferred that BDNF is effective in augmenting the recovery of neural function and capabilities related to memory, learning, and perceptual movement (87). As stated previously in this review, BNDF secretion is regulated by PGC-1α/ FNDC5/irisin. Elevated serum irisin levels induced by exercise increase BDNF levels in the hippocampus (88). Simultaneously, the modulation of FNDC5 expression in cortical neurons through siRNA resulted in the downregulation of BDNF expression (15). These studies demonstrate that irisin amplifies the neuroprotective effect of BNDF and is a pivotal factor in the early stage of brain injury.

### Irisin protects neuronal damage and prevents apoptosis by the integrin **α**V**β**5/AMPK pathway

Within 24 h of the occurrence of ICH, the levels of irisin and its receptor, integrin αVβ5, reach their peak. Subsequent treatment with irisin results in notable enhancements in both short-term and long-term neurological function, alongside a reduction of brain edema subsequent to ICH. Notably, integrin αVβ5 is predominantly localized in microglia, and the administration of irisin effectively suppresses the pro-inflammatory polarization of microglia/macrophages while facilitating their anti-inflammatory polarization. Moreover, the administration of irisin effectively hindered neutrophil infiltration following ICH and mitigated the apoptosis of neuronal cells. Mechanistically, irisin notably upregulates the expression of integrin αVβ5, p-AMPK (phosphorylated adenosine phosphate activated protein kinase), and Bcl-2 (proteins of the B-cell lymphoma-2), while downregulating the expression of IL-1 β, TNF-α, MPO (myeloperoxidase), and Bax (Bcl-2-associated X protein). This study by Wang et al. provides evidence that irisin treatment ameliorates neurological dysfunction, reduces brain edema, and alleviates neuroinflammation and neuronal apoptosis through the integrin αVβ5/AMPK signaling pathway (89).

### Irisin effectively mitigates the permeability of the blood-brain barrier subsequent to TBI

TBI places a significant global burden in terms of its high prevalence, mortality, and morbidity rates. A crucial determinant of its unfavorable clinical prognosis is the occurrence of brain edema resulting from blood-brain barrier impairment after the injury. The potential mechanism underlying this phenomenon may be associated with the morphological and functional abnormalities of neuronal mitochondria in the affected brain tissue, a reduction in uncoupling protein 2 (UCP2) activity, and an elevation in inflammatory response and oxidative stress. Guo et al. (90) found a correlation between the extent of cerebral trauma and the concentrations of irisin in the CSF. Additionally, they discovered that both endurance exercise and the administration of irisin were successful in alleviating the impairment of the blood-brain barrier in the mouse model of brain injury. Furthermore, in the UCP2 knockout mouse model, irisin exhibited the capacity to ameliorate the impairment of mitochondrial structure and function by augmenting the expression of UCP2 on the neuronal mitochondrial membrane. This led to a reduction in inflammatory responses and oxidative stress, thereby improving the blood-brain barrier and alleviating brain edema from TBI.

In short, irisin exerts a protective effect on cognitive function following TBI by inducing BNDF expression, mitigating mitochondrial damage, promoting mitochondrial occurrence, reducing neuronal apoptosis, and preserving the blood-brain barrier.

## Conclusion

**Figure 1:**
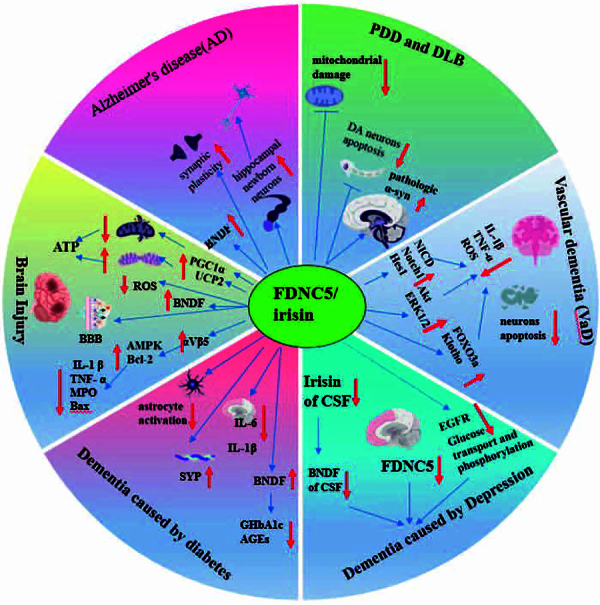
The beneficial effects of irisin on diverse forms of dementia/cognitive impairment and the underlying multiple mechanisms. PDD: Parkinson's disease with dementia; DLB: dementia with Lewy bodies.

This review provides a comprehensive overview and updated analysis of the role and mechanisms of FNDC5/irisin in different forms of dementia and cognitive impairment. Irisin demonstrates a favorable impact on nearly all forms of dementia and cognitive impairment. The potential mechanisms can be summarized as: 1) Inducing the expression of BNDF; 2) Modulating hippocampal neurons to enhance synaptic plasticity, influencing the development and maturation of newly generated hippocampal neurons, and modifying their morphology, transcription, and functionality; 3) Safeguarding the integrity of the blood-brain barrier; 4) Reducing the accumulation of pathological α-syn in the brain; and 5) Attenuating inflammation and oxidative stress levels in the nervous system, mitigating mitochondrial dysfunction, and reducing neuronal apoptosis through the involvement of Notch, Akt/ERK1/2, αVβ, and AMPK signaling pathways, and Klotho protein; and 6) Attenuating depression (Figure 1).

FNDC5/irisin is upregulated during physical activity, however, implementing regular exercise regimens for individuals with dementia and cognitive impairment may be challenging and potentially dangerous. Additionally, enhancing cognition through elevated irisin levels from exercise may not be a feasible approach in this population.

Many studies have demonstrated the ability of irisin to traverse the blood-brain barrier, which suggests that administration of exogenous irisin medication holds promise as a viable therapeutic approach for addressing dementia and cognitive impairment in the future. Nevertheless, there is a lack of comprehensive research on the utilization, dosage, and efficacy of exogenous irisin in this specific disease.

The safety and feasibility of administering irisin to humans are still uncertain, and additional clinical research is needed to establish and evaluate its potential efficacy in the treatment of dementia and cognitive impairment.
